# Influence of Cytokinins in Combination with GA_**3**_ on Shoot Multiplication and Elongation of Tea Clone Iran 100 (*Camellia sinensis* (L.) O. Kuntze)

**DOI:** 10.1155/2014/943054

**Published:** 2014-01-28

**Authors:** Reza Azadi Gonbad, Uma Rani Sinniah, Maheran Abdul Aziz, Rosfarizan Mohamad

**Affiliations:** ^1^Department of Crop Science, Faculty of Agriculture, Universiti Putra Malaysia (UPM), 43300 Serdang, Malaysia; ^2^Department of Seed and Plant Improvement, Tea Research Institute (TRI), Guilan 44159-77555, Iran; ^3^Department of Agriculture Technology, Faculty of Agriculture, Universiti Putra Malaysia (UPM), 43300 Serdang, Malaysia; ^4^Department of Bioprocess Technology, Faculty of Biotechnology and Biomolecular Sciences, Universiti Putra Malaysia (UPM), 43300 Serdang, Malaysia

## Abstract

The use of *in vitro* culture has been accepted as an efficient technique for clonal propagation of many woody plants. In the present research, we report the results of a number of experiments aimed at optimizing micropropagation protocol for tea (*Camellia sinensis* (L.) O. Kuntze) (clone Iran 100) using nodal segments as the explant. The effect of different combinations and concentrations of plant growth regulators (PGR) (BAP, TDZ, GA_3_) on shoot multiplication and elongation was assessed. The influence of exposure to IBA in liquid form prior to transfer to solid media on rooting of tea microshoots was investigated. The results of this study showed that the best treatment for nodal segment multiplication in terms of the number of shoot per explant and shoot elongation was obtained using 3 mg/L BAP in combination with 0.5 mg/L GA_3_. TDZ was found to be inappropriate for multiplication of tea clone Iran 100 as it resulted in hyperhydricity especially at concentrations higher than 0.05 mg/L. Healthy shoots treated with 300 mg/L IBA for 30 min followed by transfer to 1/2 strength MS medium devoid of PGR resulted in 72.3% of shoots producing roots and upon transferring them to acclimatization chamber 65% survival was obtained prior to field transfer.

## 1. Introduction

Tea is the most consumed nonalcoholic drink next to water in the world. The earliest records of tea date back to five thousand years ago in China, and since then it has been utilized both as a beverage and as a medicine. The powerful antioxidant properties of tea are generally attributed to its flavonoid components: theaflavins, bisflavanols, and theaflavic acids. These compounds possess potent antioxidant activity and, when consumed, act as the free radical scavengers [1]. Tea is one of the important crops in Iran covering a land of 34,000 hectares [2]. Like many other woody plant species, propagation through seeds is not desirable due to heterozygosity resulting in variations amongst the seedlings produced. Vegetative propagation using cuttings is currently the main method used but this method is hindered by the low survival of the cuttings as well as the time taken for rooting. A suitable *in vitro* propagation method would remove many of the problems related to the rooting of woody stem cuttings and also ensure the production of genetically uniform plants. There are a number of published studies describing successful protocols for micropropagation of tea [3–7]; however, thus far no research has been carried out specifically on micropropagation of Iranian tea clones. Various clones have been established in Iran, and research has shown that tea clone Iran 100 has high yields as well as high chemical contents making this clone a suitable candidate for dry tea production [8]. This study aims to utilize the available information in the literature to optimize a method for tea clone Iran 100. Plant growth regulators especially BAP and TDZ have been implicated for multiplication of tea shoots *in vitro* with response varying based on clones. According to Borchetia et al. [7] BAP at the concentration 4 mg/L was advantageous for tea clone TV25 which resulted in 5.4 shoot per nodal segment while in another study using a different tea clone, TDZ appeared superior to BAP in giving high number of shoots (8.1 shoot per nodal segment) [9]. The above mentioned statement clearly shows that response to different types of cytokinins can be specific to clones. Upon optimization of the multiplication step, elongation of shoots is necessary prior to rooting in tea. Generally, multiplication and elongation in tissue culture are done in two separate stages. However, in order to reduce the time taken and to minimize the cost of plantlet production, the multiplication and elongation stages were combined in this experiment by using the appropriate combination of different types and concentrations of cytokinins with GA_3_. Thus, the objective of this study was to develop a rapid and efficient micropropagation protocol using nodal segments for tea clone Iran 100. In addition, the ability to root and survive after acclimatization is also reported.

## 2. Materials and Methods

### 2.1. Sterilization and Induction of Nodal Segments

Nodal segments measuring 1–1.5 cm in length was excised from shoot cuttings of tea (*Camellia sinensis *(L.) O. Kuntze) plants of clone Iran 100. They were then soaked in Tween 20 for 15 minutes followed by surface sterilization with 20% Clorox solution (0.53% sodium hypochlorite) for 15 minutes. All traces of Clorox solution were then washed off with sterile distilled water. Cultures were initiated on Murashige and Skoog [10] medium supplemented with 1 mg/L BAP and 3% sucrose (w/v) and solidified using agar (0.8%; w/v) for induction of shoots. After 30–35 days of inoculation the *in vitro* shoots were transferred to media containing BAP or TDZ in combination with GA_3_.

### 2.2. Shoot Multiplication

Shoots obtained from explants initiated *in vitro* from field were used for further multiplication of shoots. The media for multiplication was MS medium supplemented with BAP at 0, 1, 3, 5, or 7 mg/L or TDZ at 0, 0.025, 0.05, 0.075 or 0.1 mg/L in combination with GA_3_ at 0, 0.5, or 1 mg/L. After 4 weeks, the mean number of shoots induced per explant was counted. In addition the fresh weight of explants produced from a single shoot was measured, and they were then transferred to fresh media of the same constituent, and their multiplication rates were evaluated. Sub culturing was done at regular intervals of four weeks up to 16 weeks (four subcultures). Number of shoots per explant after each subculture was noted.

### 2.3. Rooting and Acclimatization

For rooting, shoots (2-3 cm in height) were subjected to treatment with IBA solution (0, 100, 300, and 500 mg/L) for 30 minutes and then transferred to half-strength basal MS medium without growth regulators. After 60 days the percentage of rooted plant was noted and healthy plantlets with 4–6 leaves and well-developed roots were washed in distilled water and transferred to glass pots (5 × 12 cm) containing vermiculite and maintained in the culture room at 28°C day/20°C night, 16 h day length, and 70% humidity. Humidity was maintained by covering the glass pots with a plastic cover. The plastic cover was removed after 20 days. This was followed by placing the pots under the normal greenhouse conditions for further weaning. Upon initiation of 1–3 new leaves, the plantlets were transferred to a bigger pot (14 × 16 cm) containing peat and vermiculite (3 : 1) and maintained in the greenhouse. The percentage survival was recorded on the sixtieth day in greenhouse and then transfer to the field.

### 2.4. Culture Conditions

The media was prepared using the standard MS salts and the pH was set at 5.7 by adding KOH (1 N) or HCL (1 N). The media was solidified with 0.8% agar (w/v) and sterilized under the pressure of 103 kPa with 121°C for duration of 15 minutes. The explants were inoculated in culture tube (15 cm in height × 2.5 cm in diameter) which contained 20 mL of solid media and closed with a polypropylene cap. After initial establishment, the explants were transferred to 150 mL flask, containing 45 mL of solid media and capped with aluminum foil. All cultures were maintained at 60%–65% relative humidity, 25 ± 2°C, under light fluorescent daylight tubes emitting about 60 *μ*mol m^−2^ s^−1^, with a 16 h/8 h photoperiod. Subculture was carried on regular basis every four weeks.

### 2.5. Statistical Analysis

The treatment combinations for the BAP, TDZ, and GA_3_ were arranged in a randomized complete block design (RCBD) as a factorial experiment. Due to the large number of experimental units in this experiment, each replication was placed in a different shelf in culture room. Each shelf (replication) was considered as a block to exclude its effect as an independent source of variation during analysis of variance (ANOVA). This arrangement reduces the mean square of error and improves the accuracy of experiment. The experimental unit consisted of 2 explants and was replicated four times for all five subcultures. Observations on multiplication rates after each subculture were noted with respect to the number of shoots per explant, shoot length, and fresh weight. The mean number of shoots per explant for all BAP and GA_3_ combination at each subculture was pooled and used for comparing among subcultures through one way analysis of variance. Mean separation was carried out using Duncan's multiple range test to determine the best subculture. The rooting experiment was analyzed as a single factor.

## 3. Results

### 3.1. Shoot Multiplication and Elongation with Different Concentrations of BAP in Combination with GA_3_

**Figure 1:**
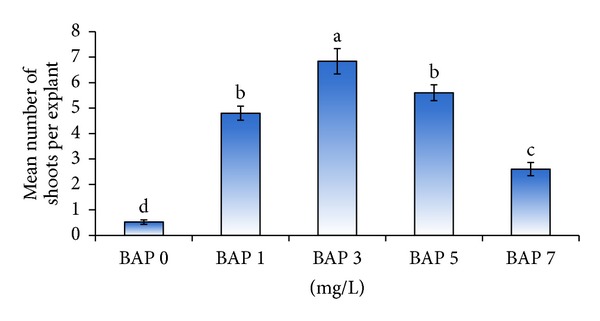
Effect of different concentrations of BAP (mg/L) on mean number of shoots per explant during shoot multiplication.

**Figure 2:**
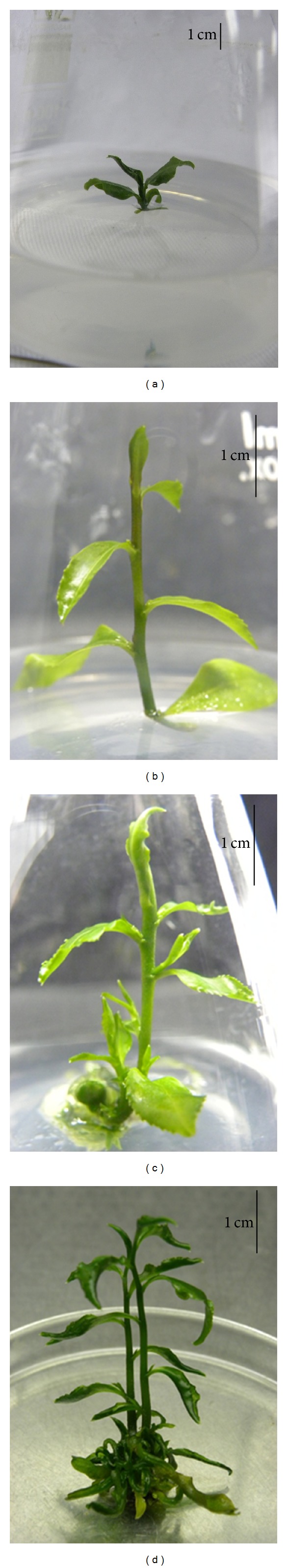
Effect of different concentrations of BAP (mg/L) in combination with GA_3_ (mg/L) on shoot multiplication and shoot elongation, (a) BAP 0 + GA_3_ 0, showing no elongation; (b) BAP 0 + GA_3_ 0.5, showing elongation of single shoot; (c) BAP 1 + GA_3_ 0.5, showing multiplication and elongation of axillary buds; (d) BAP 3 + GA_3_ 0.5 mg/L, showing multiplication and elongation of more than one shoot.

The nodal segments showed multiplication after two weeks of culture in media supplemented with all concentrations of BAP. Although variations were observed in the response of explants to plant growth regulator treatment at the earlier subculture, the results reported here reflect the status of multiplication after four subcultures, reported on the performance per shoot basis and not as cumulative output after four subcultures. In this experiment, the simple effect of GA_3_ did not show any difference in the mean number of shoots produced (data not shown), but it was observed that shoot elongation occurred when GA_3_ was included in the media (Figure 2(b)). In contrast, the results revealed that the number of shoots produced was significantly (*P* ≤ 0.01) affected by the different concentration of BAP used and the highest mean number of shoots (6.8) was obtained on MS medium supplemented with 3 mg/L BAP (Figure 1). BAP was found to be necessary for multiplication as medium with 0 mg/L BAP did not show any multiplication except for growth of single shoot. Increased concentration of BAP increased the number of shoots produced attaining a maximum number at 3 mg/L BAP but further increase in BAP concentration (5 to 7 mg/L) reduced the number of shoots produced, showed necrosis, and had shoot fasciation. The reduced number of shoots could be due to inhibition of adventitious meristem elongation due to the use of higher BAP concentration as stated by Borchetia et al. [7]. The results of analysis of variance showed that there was no significant interaction between BAP and GA_3_ combinations. Despite the lack of interaction between BAP and GA_3_ on shoot multiplication, but based on plantlet morphology there were differences, which is shown in Figure 2. The control treatment devoid of BAP or GA_3_ had no multiplication or elongation (Figure 2(a)). While the inclusion of GA_3_ into BAP free medium resulted in elongation of single shoot (Figure 2(b)). The use of 1 mg/L BAP together with 0.5 mg/L GA_3_ stimulated the growth of axillary buds while 3 mg/L BAP in combination with 0.5 mg/L GA_3_ had multiple shoots with at least two shoots with a height of around 3 cm each containing about 5 to 6 nodal segments. In relation to effective multiplication, the presence of GA_3_ induced shoot elongation resulting in prominent nodal segments which can be utilized for further multiplication during the subculture.

**Figure 3:**
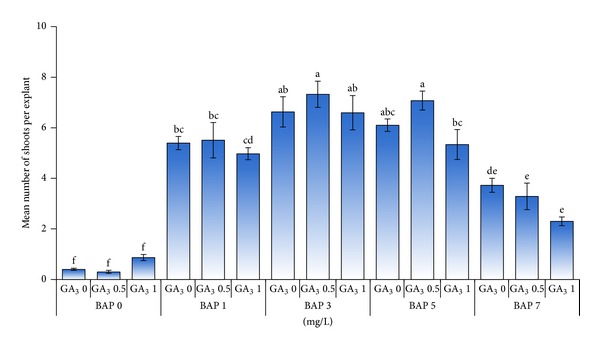
Effect of different concentrations of BAP (mg/L) in combination with GA_3_ (mg/L) on the number of shoots per explant.

Based on the above changes in the plantlet morphology due to the PGR treatments and based on the results shown in Figure 3, the best treatment for obtaining high mean number of shoots per explant (with a value of 7.3) at the fourth subculture was obtained on MS medium with 3 mg/L BAP in combination with 0.5 mg/L GA_3_. Although 5 mg/L BAP in combination with 0.5 mg/L GA_3_ also resulted in good multiplication based on number of shoots per explant, however, the plantlet morphology was not favorable.

**Figure 4:**
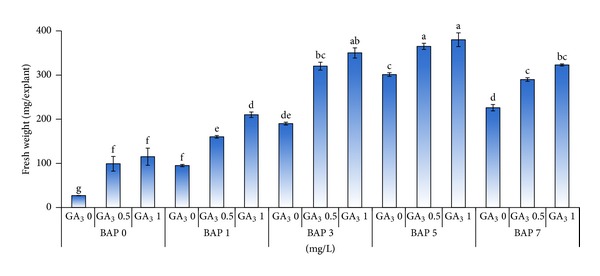
Changes in fresh weight per explant basis as affected by different concentrations of BAP (mg/L) in combination with GA_3_ (mg/L) for tea clone Iran 100.

To substantiate the data obtained on number of shoots, the fresh weight of explants produced was recorded. The results indicated highly significant differences (*P* ≤ 0.01) in fresh weight of explants among the different treatment combinations. In this study, the interaction between different concentrations of BAP in combination with GA_3_ was significant. The highest mean fresh weight per explant (380 mg) was obtained in MS media supplemented with 5 mg/L BAP in combination with 1 mg/L GA_3_ at the fourth subculture (Figure 4). As it can be seen in Figure 4, fresh weight increased with the increase in BAP concentration up to 5 mg/L due to the combined influence of increase in number of shoots as well as shoot height. At high concentration of BAP (7 mg/L) the explants favored callus formation, and this is the reason for the decrease in explant weight.

### 3.2. Multiplication of Shoots with Different Concentrations of TDZ in Combination with GA_3_

**Figure 5:**
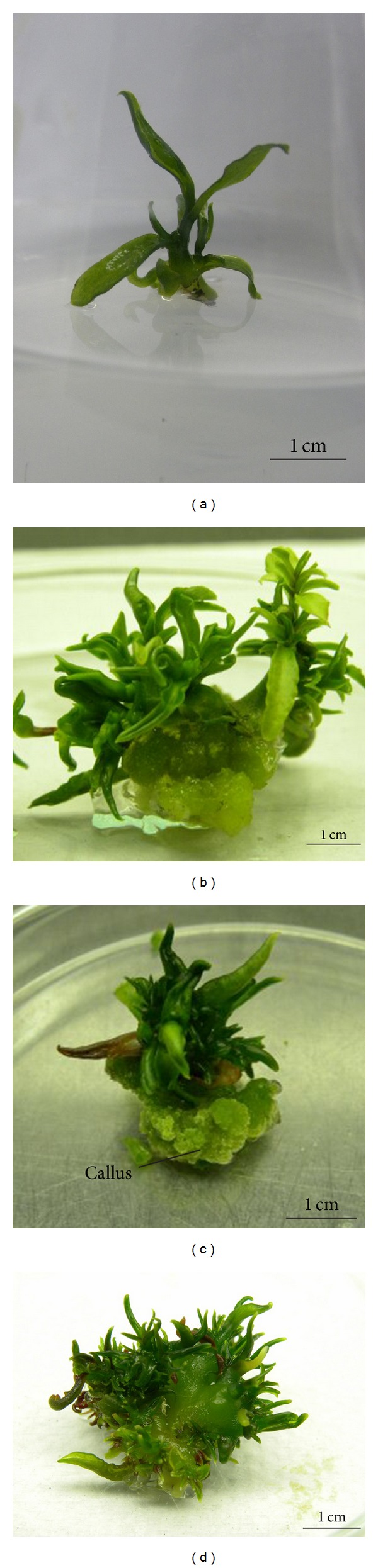
Undesirable influence of different concentrations of TDZ (mg/L) in combination with GA_3_ (mg/L) on shoot multiplication, (a) TDZ 0 + GA_3_ 0; (b) TDZ 0.025 + GA_3_ 0.5; (c) TDZ 0.075 + GA_3_ 0.5; (d) TDZ 0.1 + GA_3_ 0.

Nodal segments cultured in media which contained TDZ in combination with GA_3_ showed signs of multiplication after one week of culture except for treatment devoid of TDZ. This means that TDZ was able to show its effect faster as compared to BAP for tea clone Iran 100; however, the nodal segments were not able to sustain this pattern of growth and development during the next regular subculture. When various concentrations of TDZ ranging from 0.025 to 0.1 mg/L were used, explants multiplied in the first subculture but did not multiply in next subcultures and the explants either formed callus or became necrotic. The results indicated that the different concentrations of TDZ significantly affected the mean number of shoots per explants over subculture cycles. The highest mean number of shoots produced per explant (5.9) was obtained in MS basal media supplemented with 0.05 mg/L TDZ. Meanwhile, the lowest mean number of shoots produced per explant (0.6) was observed in the control group (MS medium without plant growth regulator). It was also observed that increasing the concentration of TDZ from 0.05 to 0.1 mg/L resulted in a decline in the mean number of shoots produced per explant from 5.9 to 1.2. Furthermore, moderate concentrations of TDZ (0.025 to 0.05 mg/L) increased the multiplication, but high concentrations of TDZ (0.075 to 0.1 mg/L) decreased multiplication and especially caused callus formation (Figure 5(c)). In addition, most of the plants growing in high concentrations of TDZ were necrotic (Figure 5(d)). In most cases the plants were hyperhydrized. Excessive callus formation, hyperhydricity and low shoot multiplication indicates that the use of BAP is more suitable than TDZ.

**Figure 6:**
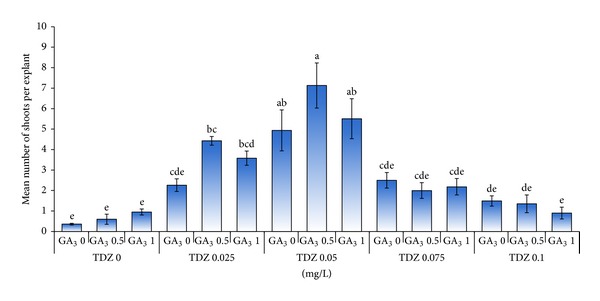
Effect of different concentrations of TDZ (mg/L) in combination with GA_3_ (mg/L) on the number of shoots per explant.

The results indicated that the highest mean number of shoots produced per explant was attained in MS media supplemented with 0.05 mg/L TDZ in combination with 0.5 mg/L GA_3_ (Figure 6). Overall, the results indicated that the mean number of shoots produced per explant decreased when the concentration of TDZ increased from 0.05 to 0.1 mg/L in combination of different concentrations of GA_3_.

**Figure 7:**
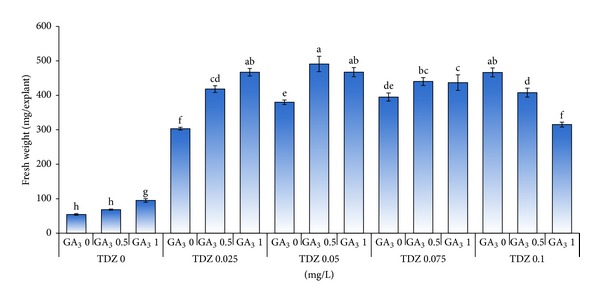
Effect of different concentrations of TDZ (mg/L) in combination with GA_3_ (mg/L) on fresh weight of shoots produced per explants of tea clone Iran 100.

Increasing the concentration of TDZ caused an increase in fresh weight of explants produced. In this experiment, the highest fresh weight was obtained on MS medium supplemented with 0.05 mg/L TDZ in combination with 0.5 mg/L GA_3_ (Figure 7). In addition, the control group (without TDZ) irrespective of GA_3_ (from 0 to 1.0 mg/L) concentrations resulted in the lowest mean fresh weight per explant.

### 3.3. Rooting and Acclimatization

**Figure 8:**
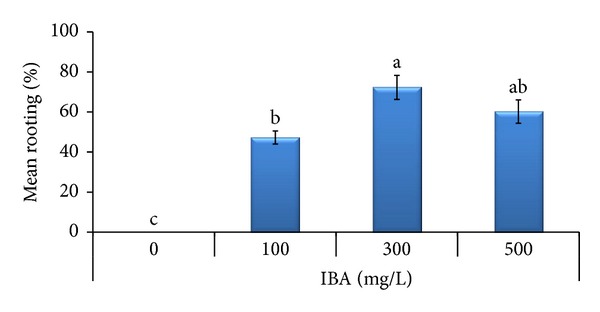
The Effect of dipping in different concentrations of IBA on percentage of rooted shoots.

**Figure 9:**
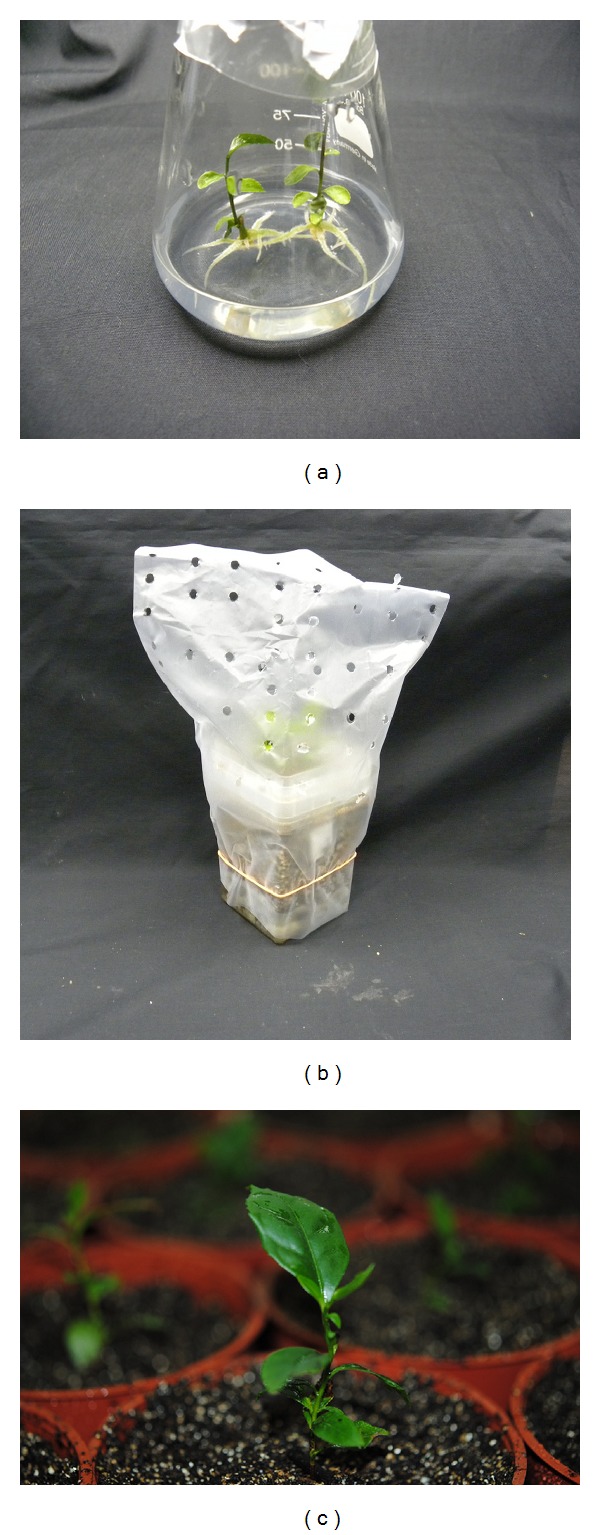
Rooting and acclimatization. (a) Rooted microshoots of tea after IBA treatment; (b) rooted cutting in vermiculite, covered with plastic for retention of humidity during acclimatization; (c) plants transferred to plastic pots in greenhouse.

The result presented in Figure 8 clearly shows the importance of IBA for rooting of tea microshoots as no root formation was observed for treatment without IBA. Root initiation required about 40 days and another 20 days for development of a root system (Figure 9(a)) of the microshoots. A high percentage of rooting (72.3%) with development of healthy roots was observed by dipping the cut ends of the shoots in the solution of 300 mg/L IBA for 30 minutes and immediately transferred to half strength MS basal medium without any added growth regulators. Increasing the IBA concentration from 300 to 500 mg/L decreased the percentage of rooting (Figure 8). Well-developed plantlets were transferred to plastic pots (Figure 9(b)) with plastic cover for four weeks and then transferred to pot (Figure 9(c)). The final percentage of survival was 65% after 60 days in green house.

## 4. Discussion

Micropropagation in tea dates back to the early 80s with numerous advancements to date [11]. However, with the development of new clones through breeding programs, response to micropropagation can differ. This paper reports on a rapid and efficient method for micropropagation of tea clone Iran 100 which incorporates the stage of multiplication and elongation into a single step protocol to reduce the cost of production. The two most commonly used cytokinins, namely, BAP and TDZ, reported to have positive effect on multiplication on different tea clones were tested.

Multiplication of primary explants is highly dependent on plant species and the type of cytokinins used. Several reports have been published on *in vitro* culture of tea and related species that showed effects of BAP [7, 12] and TDZ [13, 14] on shoot multiplication of explants. Although BAP is more commonly used, TDZ with cytokinin like activity [15] has been reported to be more potent than most of the commonly used cytokinins in tissue culture of many woody plants [16], hence the justification for testing these two plant growth regulators for tea clone Iran 100. Tea nodal segments cultured on medium with BAP or TDZ gave different response. Our result showed that TDZ was not as good as BAP because it resulted in hyperhydricity. Hyperhydricity is a phenomenon which has been reported to occur due to high concentrations of cytokinins. Its occurrence is rather species specific occurring easily in some species, such as *Lavandula vera* DC [17], *Malus sylvestris* [18], and *Vitis vinifera* [19]. In *Pyrus pyrifolia *the type of cytokinin had high influence on the formation of hyperhydrized plants while the concentration had little influence. It was also reported by these authors that synthetic phenylurea derivatives (CPPU and TDZ) produced more hyperhydric shoots than adenine derivatives (BA and kinetin) [20]. Therefore, cytokinin type and concentration suitable for micropropagation of woody plants have to be carefully optimized.

The data reported in this study is based on observation made after four subculture cycles. The observations show that continuous subculture on medium with TDZ concentration of above 0.025 mg/L resulted in hyperhydricity. It is believed that if subculture is carried out on medium devoid of TDZ upon initial culture on medium with TDZ, the problem of hyperhydricity can be curbed. Furthermore, increased callus formation and fresh weight at high concentrations of TDZ was prominent. The outcome of the present research complies with that of Mondal et al. [13] who found that while the responsive explants were progressively grown on the medium possessing TDZ, an overall callus overgrowth and later necrosis were observed. Furthermore, this finding is in accordance with that Bohmer et al. [21] and Murthy et al. [22] showing the adverse influence of constant exposure to TDZ on the multiplication and growth of pea and chickpea. The recent achievements regarding the adverse effect of TDZ can be best described by virtue of the capacity of TDZ in stimulating endogenous cytokinin biosynthesis or altering cytokinin metabolism [23, 24], and it is necessary to remove TDZ in subsequent subcultures in order to increase the multiplication rates or the sturdiness of the shoot. In contrast, the findings of the current study showed that the continuous presence of BAP is imperative for shoot multiplication media. Constant presence of BAP at optimal concentrations was necessary at different subcultures and there seemed to be a cumulative gain over subcultures in both the number as well as the sturdiness of the shoots. These results clearly showed that TDZ was inferior to BAP in clone Iran 100.

The most important characteristics of the cytokinins are to stimulate shoot multiplication and inhibit their elongation. In order to overcome the inhibition of shoot elongation, various methods were used by transferring the explants to secondary medium, supplemented with low concentrations of cytokinins or using different growth regulators [25–27]. Otherwise, keeping the explants, at low temperatures (chilling treatments) may be effective for shoot elongation [28]. In this study, a suitable plant growth regulator was selected to overcome the inhibition of cytokinins on shoot elongation.

The new challenges which are faced today by the tissue culture industry include cost efficiency, automation, control and optimization of the *in vitro* microenvironment, and so forth [29]. It is, therefore, important to bring about further improvements in the existing tissue culture protocols [30]. Hence, alternatives to expensive inputs and infrastructure have been sought and developed to reduce the costs of plant micropropagation. The composition of culture media used for shoot proliferation and rooting has a tremendous influence on production costs. The replacement of plant growth regulators used and decrease in general stages of micropropagation can reduce costs of production. In this study we tried using these combined techniques with the aim of establishing an efficient, cost saving micropropagation protocol for tea clone Iran 100. The results showed that a combination of BAP and GA_3_ unlike the use of TDZ was found to be the best for both multiple shoot formation and shoot elongation. The most intriguing outcome of the present investigation was that the aforementioned combination could have two products simultaneously. The first product is the appropriate shoots for shoot multiplication by applying BAP and the second one is multiplication of 2 to 3 cm shoots due to the use of GA_3_, which is proper for rooting.

Based on the literature on the success of rooting, there are two suitable methods for *in vitro* rooting of microshoots. They can be performed either by culturing microshoots on media with low concentrations of auxin or treating the microshoots with high concentration of auxin solution followed by transfer to an auxin-free solid medium. Usually, 1/2 strength MS medium supplemented with IBA (0.5–8 mg/ L) is used on *in vitro* rooting of tea [31, 32]. In order to improve *in vitro* rooting of tea, we treated the healthy shoots (2-3 cm height) with different high concentrations of IBA for 30 minutes before transferring to half strength MS basal medium without growth regulators. There was a significant difference between the various concentrations of IBA with 300 mg/L being the most suitable concentration resulting in 72.3% of rooted microshoots. This finding corroborates the ideas of Das et al. [33] and Jain et al. [34], who suggested treating microshoots at the cut end with high concentrations of IBA and transferring directly in the soil mix.

The mixture containing peat moss + vermiculite (3 : 1) resulted in increased percentage of plant survival of tea (clone Iran 100) and it is, therefore, a recommended medium for growth of the cultivar during acclimatization. Vermiculite possesses cation exchange properties; thus it can hold and make available ammonium, potassium, calcium, and magnesium to the growing plants. Vermiculite when combined with peat moss promotes faster root growth and gives quick anchorage to young roots [35].

## 5. Conclusions

This study established a rapid and efficient protocol for tea clone Iran 100 by combining both cytokinin for multiplication and GA_3_ for elongation. TDZ was not suitable for multiplication of this clone as it resulted in hyperhydricity and stimulated callus formation at high concentrations but BAP was found to be suitable and efficient on multiplication of shoot. One of the most important outcome from this experiment is that the combination of BAP and GA_3_ was able to provide multiplication and elongation of shoots at the same time thus reducing time and cost of production. These findings can be helpful in commercial production of the micropropagated plantlets in other plant species.

## Abbreviations

MS basal medium: Murashige and Skoog basal medium, 1962
TDZ: N-phenyl-N-1,2,3-thiadiazol-5ylurea
GA_3_: Gibberellic acid
BAP: 6-Benzylaminopurine
IBA: Indole-3-butyric acid.
